# Changes in quality of life over time through the lens of young people aged 18–24 in 2016 in Gauteng Province of South Africa

**DOI:** 10.1371/journal.pone.0338312

**Published:** 2025-12-08

**Authors:** Edmore Marinda, Yashena Naidoo, Mmakotsedi Magampa, Abongile Pindo, Mercy Ngungu, Kwanele Qonono, Lwando Mdleleni, Makgalane Ramontsha

**Affiliations:** 1 Human Science Research Council, Impact Centre, Pretoria, South Africa; 2 University of Johannesburg, SAMRC/UJ Pan African Centre for Epidemics Research (PACER), Johannesburg, South Africa; 3 Gauteng City-Region Observatory, Johannesburg, South Africa; 4 Gauteng City-Region Observatory is a Partnership of the University of Johannesburg, the University of the Witwatersrand, the Gauteng Provincial Government and Organised Local Government in Gauteng (SALGA), Johannesburg, Gauteng, South Africa,; 5 University of the Witwatersrand, Johannesburg, South Africa; University of Alabama at Birmingham, UNITED STATES OF AMERICA

## Abstract

Young people in South Africa face many challenges that include high unemployment, economic hardships, high rates of crime, alcohol and substance abuse, violence, anxiety, depression, and trauma. Quality-of-life (QoL) surveys done over time help identify the challenges faced by this group and help with designing appropriate programmes to mitigate these challenges. The study aimed to assess changes in the QoL of young people aged 18 to 24 in 2016, using a 32 to 38-year-old cohort as a comparison group. Mixed effects regression models with time and age-group interaction terms were used to assess changes in QoL between 2016 and 2024 for the two age groups using four unlinked surveys. Marginal QoL mean scores decreased from 62.5 in 2016 to 59.2 in 2024 for youth living in Gauteng province, South Africa, while for the older cohort, the mean score initially increased from 62.5 to 64.2 between 2016 and 2018 but had decreased to 60.1 by 2024. QoL scores for the older age group were marginally higher compared to the younger group in 2018 (64.2 vs 63.5), 2021 (61.6 vs 60.9), and 2024 (60.1 vs 59.2). Among youth, the following QoL domains contributed to the observed decrease in overall QoL scores: satisfaction with government, life satisfaction, health status, and satisfaction with public services, with the only noticeable consistent increase observed in the participation domain. There were noticeable increases in the marginal mean scores for the socio-economic status domain for the older cohort after the COVID-19 epidemic, with the opposite observed among the young age group. Young people in the Gauteng province of South Africa are generally discontented and dissatisfied with life as they age. Challenges with unemployment, safety, violence, and mental health need to be urgently addressed to avoid possible unrest, as observed before and elsewhere in the country.

## Introduction

South Africa is a middle-income country, yet it ranks as the most unequal societies in the world, with a Gini coefficient of 0.63 [1]. These extreme levels of inequality are rooted in the enduring legacy of apartheid, which continues to shape economic structures and access to opportunities. Economic advancement remains heavily skewed toward a small segment of the population, while the majority particularly Black Africans, who make up 82% of the population face systemic deprivation [2,3].

Over the past three decades of democratic governance, South Africa has made significant strides in improving access to education, clean water, energy, and housing. According to the 2022 South Africa national censes results, among all households, 91% had piped water, 71% had flush toilets, 68% had refuse removal services, while 95% of households had access to electricity [4]. However, systemic challenges persist particularly high levels of unemployment, crime, corruption, and stark regional inequalities [5–7]. These disparities have driven sustained urban migration, especially to Gauteng and the Western Cape province, which experienced population growth of 31% and 24% respectively between 2011 and 2022, putting major strains on infrastructure and service delivery in these two provinces [8,9]. Political instability, governance failures, and widespread corruption have eroded public trust in institutions [10,11].

Gauteng province, South Africa’s economic engine, has experienced consistent population growth due to high birth rates and both internal and cross-border migration [12,13]. However, this demographic growth has not been matched by proportional economic growth or investment in essential public services such as housing, healthcare, water, sanitation, education, and transportation. Economic opportunities in the province are not experienced homogeneously across different socio-demographic groups [14]. Moreover, the province remains shaped by spatial, economic, and social inequalities inherited from apartheid-era policies, including segregated urban planning, unequal education systems, and restricted access to quality employment [15–17]. These structural challenges continue to impact the quality of life (QoL) for different socio-demographic groups.

Young people in South Africa are confronted with a complex web of intersecting challenges that include economic exclusion, high levels of crime, substance abuse, violence, and deteriorating mental health [18]. South Africa is considered a young country demographically, with close to one-third of its population aged between 15 and 34 years [19]. Despite this potential demographic dividend advantage, youth participation in the labour market remains low. In the first quarter of 2024, 46% of youths were unemployed and this compared to a national average of 33% across all working age groups [18]. Even before the COVID-19 pandemic, the national economy was underperforming, averaging below 2% annual growth, and struggling to absorb a growing labour force [20]. Despite achieving higher levels of education than previous generations, where in 2022, 37% of Black youths, 15–34 years old completed at least five years of high school (Grade 12), compared to 17% in 2016 of the same age group achieving similar education levels, young people continue to face significantly higher levels of unemployment and economic insecurity [5,6,21]. For young people, these challenges are particularly severe, manifesting in persistent unemployment, exposure to crime, deepening inequality, limited access to quality education, and rising mental health concerns [6,22,23]. Financial insecurity and anxiety about the future are part of daily life for many. These multifaceted stressors severely impact young people’s quality of life.

Understanding and improving Quality of Life (QoL) has become increasingly central to social research and policy development especially in the context of South Africa, where structural inequality deeply shapes everyday experiences. While traditional development indicators such as Gross Domestic Product (GDP) per capita or employment rates offer important macroeconomic insights, they often fall short of capturing the lived realities, well-being, and subjective experiences of individuals and communities [24,25]. Subjective indicators such as life satisfaction, perceived safety, or trust in institutions are essential in complementing objective metrics because they reflect how people interpret their socio‑economic environments, which can diverge significantly from what income or service availability alone indicators portray [25,26]. Incorporating both dimensions enables a richer, more people-centred approach to social analysis and policymaking. For young people who are disproportionately affected by poverty, unemployment, and violence these measures reveal critical insights into resilience, hope, and frustration, which often remain unseen in conventional indicators.

### Theoretical framework

The World Health Organization defines QoL as “an individual’s perception of their position in life in the context of the culture and value systems in which they live and in relation to their goals, expectations, standards and concerns” [27].

Several theoretical frameworks, that include Maslow’s Hierarchy of Needs Theory, Carol Ryff’s Psychological Well-Being Model, Lawton’s Quality of Life Theory, the Sen’s Capabilities Approach and the Schalock’s Multidimensional Approach among others have been proposed as theoretical frameworks that underpin the understanding of QoL and well-being [28–32]. This study is closely aligned to Schalock’s multidimensional Approach, which emphasises the interconnectedness of psychological, social, material and health related aspects of wellbeing and thus inform QoL. The quality-of-life index derived and used in QoL surveys by the Gauteng City Region Observatory (GCRO) and used in this study have evolved from an initial set of 10 dimensions from 58 variables in the 2009 survey to 7 dimensions from 33 variables in the latest survey [33]. Similarly the Schalock model has 8 dimensions, a physical dimension, that includes physical health, a psychological dimension that includes emotions and behaviour, a social dimension that captures relationships and community, an environmental dimension that include home, work and society, a spiritual dimension that captures beliefs and values, an economic dimension that includes income and employment, a political dimension that include rights and involvement in decision-making processes and a cultural dimension that include cultural identity, beliefs and traditions [32]. These dimensions are very similar and overlap with our study’s dimensions that include access to services, socio-economic conditions, satisfaction and trust in government, health, safety, satisfaction with one’s life and participation in public processes. This framework emphasizes that individuals exist within interconnected social, economic, political, and institutional settings where dysfunction in one dimension may lead to cascading effects across others.

Tracking QoL outcomes among youth is therefore crucial not only to determine whether life is improving, but also to identify which specific dimensions or aspects are improving. In Gauteng province, the Quality of Life (QoL) Surveys, first conducted in 2009, offers an invaluable resource of panel data on both objective and subjective well-being dimensions [34]. Since 2009, seven iterations of the Gauteng provincial QoL surveys have been conducted, and this paper make use of four surveys undertaken between 2016 and 2024. These four surveys were used because they had standardized questions and similar dimensions, which enable reliable trend analysis.

### Objectives

The main objective of this study was to assess whether the QoL of young people in Gauteng province improved between 2016 and 2024, comparing them to a reference group of adults aged 32–38 years old in the same province. In addition to the overall QoL scores, the seven dimensions that made up the overall QoL scores were also assessed for the two cohorts. A secondary objective was to assess the impact of COVID-19 on the QoL scores among the two cohorts in Gauteng province.

## Methods

This paper explores trends in QoL scores and associated QoL dimensions over an eight year period, 2016–2024, tracking and assessing QoL scores for young people who were aged 18–24 years old in 2016, referred to as age-cohort-1 from hereon, using four unlinked cross-sectional QoL surveys done in 2016, 2018, 2021 and 2024 in the Gauteng province of South Africa. An older age cohort aged 32–38 years old in 2016, referred to as age-cohort-2 from hereon, was used as a comparison group to see if QoL differed by age cohort over time. The study followed these age cohorts, assessing their QoL scores as they aged over the eight-year period. Data for the study came from randomly selected individuals aged 18 years and older residing in Gauteng province at the time of the surveys. Data for the 2016, 2018 and 2021 were downloaded on the 19^th^ of July 2024, while data for 2024 was accessed on the 31 of December 2024. All survey data is available on the Gauteng City Region Observatory (GCRO) Website https://gcro.ac.za/research/research-themes/detail/quality-life/.

Ethics clearance for the study was obtained from the University of the Witwatersrand (H19/11/09). Informed consent was obtained from all study participants. All data is anonymous with no identifiers that can be used to identify survey participants.

### Variables

**Fig 1:**
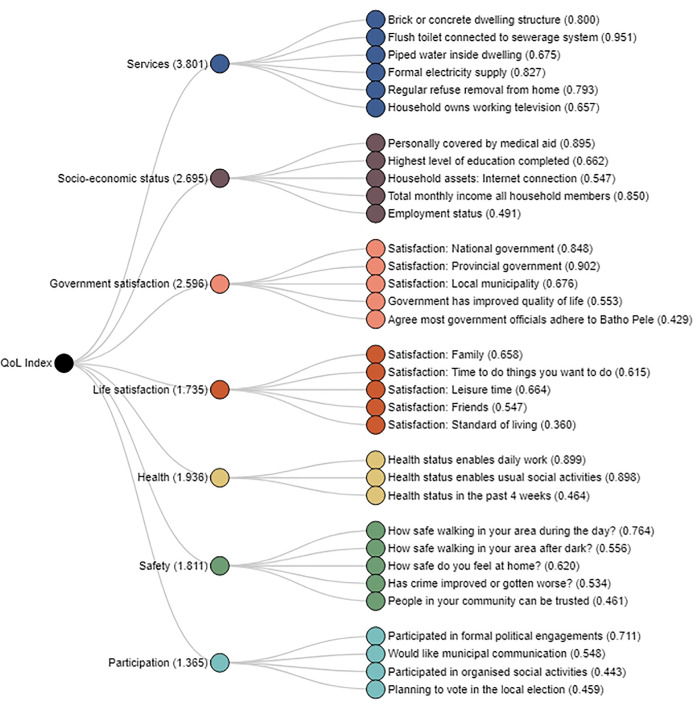
Variables making up each of the seven dimensions of the QoL Index. Note that factor loadings are included brackets, while for dimensions, the eigenvalues are included.

The QoL index is a composite multi-dimensional index made up of weighted domains that cover health, education, everyday activities such as work, housing, participation in political processes, social relationships, safety from bodily harm and the environment in which people live [35]. For the four GCRO QoL surveys used in this study, Quality of Life scores were constructed from the following seven dimensions: services, socioeconomic status, satisfaction with life, satisfaction with government (national, provincial, and municipality), health status, safety, and participation. Each dimension was constructed from a set of questions ranging from three to six questions, Fig 1 [33,36]. In brief the QOL index was derived from 33 variables making seven dimensions, which were aggregated into a single score out of 100. Exploratory factor analysis (EFA) using the 2017/18 survey dataset, and validation through confirmatory factor analysis (CFA) using the 2017/18, 2015/16 and the 2013/14 surveys was used to select variables and construct dimensions. Full details of how the QoL scores for each study were constructed are described elsewhere [33,37]. QoL scores and the seven dimensions were extracted from the 2016, 2018, 2021, and 2024 surveys data for the two age-cohort groups as described above. Also extracted from the four rounds of survey datasets were socio-demographic variables that included sex, race, whether one was in a relationship or not, household size, highest education attained, migration status, whether anyone in the household received a government social grant or not, total household income, type of housing dwelling, ownership of the house dwelling respondent lived in, and whether respondents sent remittances to family or relatives.

### Sampling

Sampling of survey participants involved several steps. Firstly, all residential buildings in the province were identified using aerial mapping using Building Land-use dataset from GeoTerraImage [38]. All identified residential buildings that included free standing houses, hostels, apartments blocks, and informal dwellings where then grouped according to the geographic ward and/or enumeration area that they belonged to [39]. From each ward, a minimum prescribed number of interview locations, based on each year’s sample size and stratification were then randomly selected. Fieldworkers then visited the selected interview locations, and once they were within 80 meters of the target location using electronic maps, they selected a dwelling unit to conduct the interview. On instances where there were more than one dwelling unit at a location, the software on the tablet was used to randomly select a unit. Thereafter, the fieldworker listed all adults, 18 years or older, living in that dwelling unit, and they then randomly selected a person to interview using their tablet. Data was collected and captured in real time and quality assured as it came through.

### Data management and data analysis

Data was imported and analysed in STATA 18 (STATA Corp). Categorical variables were reported as frequencies and percentages, while means and standard deviations (sd) were reported for QoL scores and their dimensions.

Mixed effects regression models comparing the two age groups were fitted to assess changes in QoL (the outcome/dependent variable of interest), over the eight-year study period. Firstly, a uni-variable mixed effects regression model fitting time, with 2016 as the reference year and dummies for 2018, 2021, and 2024, and age cohorts 1 (18–24 years), and 2(32–38 years) as the reference group were fitted with an interaction term between these two variables (time indicator and age cohort) and district as the random variable. Uni-variable models for each socio-demographic factor were also fitted on the QoL score variable. A multi-variable mixed effects model with time-by-age-cohort variables and an interaction term for these two variables, adjusting for socio-demographic variables, and including district as a random effect was then fitted. Expected marginal mean scores without the random component of district were then estimated for each time by age cohort group and plotted over time to aid with the easier interpretation of the results. To explore the contribution of each QoL dimension over time, the above estimation steps were repeated for each dimension score, and expected marginal mean scores were estimated and plotted. Regression coefficients were reported with 95% confidence intervals and p-values. Statistical significance was assumed at 5%.

Survey weights to benchmark age, sex, race, and district-estimated populations in that year were used for all inferences. Sensitivity analysis was carried out to assess the effect of larger sample sizes in the first two surveys (2016 and 2018), with new weights derived by adjusting down the weights for 2016 and 2018 and adjusting up the weights for 2021 and 2024.

## Results

### Study sample

Twenty-six thousand, three hundred and fifty-seven (26,357) individual records were extracted from the four QoL surveys done by the GCRO, Table 1. These included 10 282 individuals in 2016 who were aged 18–24 (age-cohort-1) and 32–38 (age-cohort-2), 8 603 individuals in 2018 who were now aged 20–26 (age-cohort-1, i.e., the group that was 18–24 in 2016) and 34–40 (age-cohort-2, i.e., the group that was aged 32–38 in 2016), 4 008 individuals in 2021 who were aged 23–29 (age-cohort-1, i.e., the group that was aged 20–26 in 2018) and 37–43 (age-cohort-2, i.e., the group that was 34–40 in 2018), and 3 464 individuals in 2024 who were aged 26–32 (age-cohort-1, i.e., the group that was aged 23–29 years old in 2021) and those aged 40–46 (age-cohort-2, i.e., the group that was aged 37–43 in 2021). Of the total sample, 14,007 were age-cohort-1 participants, and 12,350 were age-cohort-2 participants.

**Table 1:** Sample Size for QoL tracking study for young people aged 18 to 24 in Gauteng, 2016 - 2024.

		Age cohort group		
		18-24yrs	32-38yrs	Total
		n	n	n
	2016	4 674	5 608	10 282
**Year**	2018	4 692	3 911	8 603
	2021	2 487	1 521	4 008
	2024	2 154	1 310	3 464
	Total	14 007	12 350	26 357

The sample sizes for 2016 and 2018 were much larger than the other two survey rounds because there was more funding that enabled more granular reporting. Since these are unlinked cross-sectional surveys, some individuals may have participated in multiple surveys over time. There was however no way of ascertaining this information from the surveys because no data was collected from individuals to indicate if they had participated in any previous surveys. Details of sample sizes and sampling for each of the four surveys are described elsewhere [34].

### Socio-demographic factors

Table 2 shows the socio-demographic characteristics of survey participants by age group over the four rounds of surveys. The percentage of males participating in the survey across the four rounds of surveys and cohort groups remained relatively similar, ranging between 46% and 50%. The 2024 survey shows a small decline in the percentage of Black African participants making up the study sample compared to contributions in other years for both cohorts. The percentage of survey participants with higher qualifications increased over time for each moving cohort, i.e., whilst 17% of age-cohort-1, 18–24-year-olds in 2016 participants held a post-matric certificate (matric is five years of high school in South Africa) or had a graduate or post-graduate qualification in 2016, the percentage had increased to 34% of participants with these same qualifications in 2024. For age cohort 2, 26% and 28% had post-matric certificates or graduate or post-graduate qualifications in 2016 and 2024, respectively. Over the years, unemployment increased from 42% in 2016 to 52% in 2018 before coming down in the consecutive periods to 42% in 2022 and 30% in 2024 for age cohort-1. Similarly in age cohort-2, unemployment increased from 34% in 2016, remaining at similarly high levels of 42% in 2018 and 2022 before coming down to 33% in 2024. In age-cohort-1, the percentage of participants reporting that at least one person in their household was receiving a social grant was highest in 2016 (44%) but was down to 35% in 2024. The percentage reporting a household member receiving a grant in the age-cohort-2 group remained relatively stable, ranging between 36% and 38% over the four rounds of the surveys. Reported total household income increased noticeably among the age-cohort-1 households, with 8% reporting a total monthly household income of R12 801 (US 731) or more in 2016, increasing to 23% in 2024, possibly indicating that some of these participants had entered the labour market. Similarly, 14% in 2016 and 19% in 2024 of age-cohort-2 participants reported a total household income of R12 801 or more. Sixty-one per cent (61%) in 2016, 46% in 2018, 49% in 2021, and 58% in 2024 of age-cohort-1 participants said the house they lived in was their family home. This compared to 53% in 2016, 58% in 2018, 65% in 2022, and 72% in 2024 among age-cohort-2 participants. The percentage of age-cohort-1 participants reporting staying in informal housing ranged from 12% in 2022 to 15% in 2018, and from 10% in 2024 to 16% in 2016 among the age cohort 2 participants. Among age-cohort-1 participants, 30% in 2016, 25% in 2018, 40% in 2021, and 41% in 2024 said they sent remittances to family members. This compared to 20% in 2016, 14% in 2018, 35% in 2021, and 28% in 2024 among age-cohort-2 participants who indicated the same.

**Table 2:** Socio-demographic factor for two cohorts aged 18-24 and 32-38 in 2016 in Gauteng.

		Cohort																
		18-24 year old in 2016 group										32-38 year old in 2016 group						
		2016		2018		2022		2024		2016		2018		2022		2024		
**Sex**	**Frequency**	**%**	**Frequency**	**%**	**Frequency**	**%**	**Frequency**	**%**	**Frequency**	**%**	**Frequency**	**%**	**Frequency**	**%**	**Frequency**	**%**	**Total**	**%**
Male	2168	46,4	2168	46,2	1168	47	1108	51,4	2672	47,6	1949	49,8	713	46,9	650	49,6	12596	47,8
Female	2506	53,6	2524	53,8	1319	53	1046	48,6	2936	52,4	1962	50,2	808	53,1	660	50,4	13761	52,2
Total	4674	100	4692	100	2487	100	2154	100	5608	100	3911	100	1521	100	1310	100	26357	100
**Race Group**																		
Black African	4056	86,8	4280	91,2	2138	86	1370	81,5	4628	82,5	3171	81,1	1177	77,4	642	71	21462	84,2
Coloured	177	3,8	127	2,7	59	2,4	83	4,9	210	3,7	180	4,6	44	2,9	48	5,3	928	3,6
Indian/Asian	72	1,5	45	1	33	1,3	46	2,7	115	2,1	82	2,1	29	1,9	31	3,4	453	1,8
White	352	7,5	224	4,8	250	10,1	181	10,8	616	11	464	11,9	271	17,8	183	20,2	2541	10
Other	17	0,4	16	0,3	7	0,3	0	0	39	0,7	14	0,4	0	0	0	0	93	0,4
Total	4674	100	4692	100	2487	100	1680	100	5608	100	3911	100	1521	100	904	100	25477	100
**Highest Education attained**																		
No education	18	0,4	25	0,5	16	0,6	19	0,9	44	0,8	61	1,6	31	2	30	2,3	244	0,9
Primary	108	2,3	60	1,3	52	2,1	67	3,1	195	3,5	133	3,4	92	6	120	9,2	827	3,1
Some secondary	1446	30,9	1208	25,7	829	33,3	649	30,1	1774	31,6	1210	30,9	541	35,6	473	36,1	8130	30,8
Matric	2215	47,4	2012	42,9	824	33,1	675	31,3	2026	36,1	1155	29,5	414	27,2	315	24	9636	36,6
Certificate/Diploma	646	13,8	974	20,8	500	20,1	445	20,7	983	17,5	835	21,4	266	17,5	209	16	4858	18,4
Graduate	109	2,3	246	5,2	126	5,1	153	7,1	311	5,5	291	7,4	85	5,6	85	6,5	1406	5,3
Post graduate	42	0,9	102	2,2	127	5,1	139	6,5	180	3,2	156	4	82	5,4	70	5,3	898	3,4
Not Specified	90	1,9	65	1,4	13	0,5	7	0,3	95	1,7	70	1,8	10	0,7	8	0,6	358	1,4
Total	4674	100	4692	100	2487	100	2154	100	5608	100	3911	100	1521	100	1310	100	26357	100
**Age Cohort**																		
18-24yrs	4674	100	4692	100	2487	100	2154	100	0	0	0	0	0	0	0	0	14007	53,1
32-38yrs	0	0	0	0	0	0	0	0	5608	100	3911	100	1521	100	1310	100	12350	46,9
Total	4674	100	4692	100	2487	100	2154	100	5608	100	3911	100	1521	100	1310	100	26357	100
**Relationship status**																		
In a relationship	3113	66,6	3688	78,6	2167	87,1	1670	77,5	4634	82,6	3234	82,7	1232	81	823	62,8	20561	78
Not in a relationship	1561	33,4	1004	21,4	320	12,9	484	22,5	974	17,4	677	17,3	289	19	487	37,2	5796	22
Total	4674	100	4692	100	2487	100	2154	100	5608	100	3911	100	1521	100	1310	100	26357	100
**Household Size**																		
One	456	9,8	1203	25,6	513	20,6	427	19,8	992	17,7	872	22,3	305	20,1	357	27,3	5125	19,4
Two	731	15,6	1007	21,5	386	15,5	309	14,3	1085	19,3	571	14,6	289	19	300	22,9	4678	17,7
Three	904	19,3	849	18,1	440	17,7	371	17,2	1104	19,7	698	17,8	277	18,2	217	16,6	4860	18,4
Four	772	16,5	647	13,8	480	19,3	479	22,2	1030	18,4	852	21,8	273	17,9	195	14,9	4728	17,9
Five	704	15,1	423	9	319	12,8	293	13,6	649	11,6	483	12,3	169	11,1	118	9	3158	12
Six or more	1107	23,7	563	12	349	14	275	12,8	748	13,3	435	11,1	208	13,7	123	9,4	3808	14,4
Total	4674	100	4692	100	2487	100	2154	100	5608	100	3911	100	1521	100	1310	100	26357	100
**Social Grant Household**																		
No	2632	56,3	2939	62,6	1491	60	1348	62,6	3630	64,7	2476	63,3	967	63,6	814	62,1	16297	61,8
**Yes**	2042	43,7	1753	37,4	996	40	806	37,4	1978	35,3	1435	36,7	554	36,4	496	37,9	10060	38,2
Total	4674	100	4692	100	2487	100	2154	100	5608	100	3911	100	1521	100	1310	100	26357	100
**Employment Status**																		
Unemployed	1946	41,6	2434	51,9	1051	42,3	648	30,1	1903	33,9	1628	41,6	644	42,3	435	33,2	10689	40,6
Student	1170	25	154	3,3	16	0,6	2	0,1	45	0,8	3	0,1	1	0,1	1	0,1	1392	5,3
Retired/pension	47	1	5	0,1	2	0,1	2	0,1	43	0,8	23	0,6	18	1,2	107	8,2	247	0,9
Business owner	143	3,1	339	7,2	72	2,9	78	3,6	355	6,3	432	11	52	3,4	51	3,9	1522	5,8
Part-time employed	503	10,8	497	10,6	318	12,8	266	12,3	739	13,2	396	10,1	176	11,6	142	10,8	3037	11,5
Full time employed	865	18,5	1263	26,9	1028	41,3	1158	53,8	2523	45	1429	36,5	630	41,4	574	43,8	9470	35,9
Total	4674	100	4692	100	2487	100	2154	100	5608	100	3911	100	1521	100	1310	100	26357	100
**Household monthly income**																		
No Income	226	4,8	254	5,4	25	1,1	4	0,2	184	3,3	125	3,2	24	1,9	2	0,2	844	3,3
R1 - R1600	924	19,8	629	13,4	625	27,6	187	8,7	952	17	481	12,3	328	25,3	158	12,1	4284	16,5
R1601 - R6400	1151	24,6	1374	29,3	657	29	561	26	1374	24,5	960	24,5	331	25,6	394	30,1	6802	26,3
R6401 - R12800	340	7,3	439	9,4	232	10,2	241	11,2	440	7,8	333	8,5	134	10,4	114	8,7	2273	8,8
R12801 - R19200	157	3,4	193	4,1	127	5,6	140	6,5	280	5	164	4,2	70	5,4	73	5,6	1204	4,6
R19201 - R51200	185	4	194	4,1	211	9,3	252	11,7	388	6,9	302	7,7	134	10,4	112	8,5	1778	6,9
R51201 or more	46	1	29	0,6	68	3	94	4,4	95	1,7	69	1,8	57	4,4	50	3,8	508	2
Prefer not to say	723	15,5	1580	33,7	247	10,9	600	27,9	1446	25,8	1477	37,8	169	13,1	373	28,5	6615	25,5
Don’t know	922	19,7	0	0	76	3,4	75	3,5	449	8	0	0	47	3,6	34	2,6	1603	6,2
Total	4674	100	4692	100	2268	100	2154	100	5608	100	3911	100	1294	100	1310	100	25911	100
**Housing status**																		
Owned	2829	60,5	2138	45,6	1215	48,9	1240	57,6	2943	52,5	2264	57,9	988	65	949	72,4	14566	55,3
Renting	1172	25,1	1749	37,3	844	33,9	635	29,5	1781	31,8	1045	26,7	294	19,3	208	15,9	7728	29,3
Allowed to stay rent free by owner	359	7,7	415	8,8	255	10,3	164	7,6	517	9,2	296	7,6	149	9,8	93	7,1	2248	8,5
Squatting or living rent-free in an informal dwelling youve built, or in a vacan	173	3,7	202	4,3	140	5,6	86	4	206	3,7	179	4,6	65	4,3	49	3,7	1100	4,2
Other	141	3	19	0,4	31	1,2	25	1,2	161	2,9	15	0,4	23	1,5	10	0,8	425	1,6
Dont know	0	0	169	3,6	2	0,1	4	0,2	0	0	112	2,9	2	0,1	1	0,1	290	1,1
Total	4674	100	4692	100	2487	100	2154	100	5608	100	3911	100	1521	100	1310	100	26357	100
**Type of House**																		
House, brick or concrete	3171	67,8	2371	50,5	1453	58,4	1365	63,4	3405	60,7	2286	58,5	1072	70,5	992	75,7	16115	61,1
Room/Flat or tent	264	5,6	770	16,4	275	11,1	179	8,3	413	7,4	514	13,1	109	7,2	70	5,3	2594	9,8
Informal	701	15	834	17,8	467	18,8	336	15,6	899	16	558	14,3	196	12,9	122	9,3	4113	15,6
Complex/Estate	538	11,5	717	15,3	292	11,7	274	12,7	891	15,9	553	14,1	144	9,5	126	9,6	3535	13,4
Total	4674	100	4692	100	2487	100	2154	100	5608	100	3911	100	1521	100	1310	100	26357	100
**Place of birth**																		
Born in Gauteng	3236	69,2	2527	53,9	1181	47,5	1019	47,3	3316	59,1	2098	53,6	767	50,4	757	57,8	14901	56,5
Migrated other province	1161	24,8	1653	35,2	964	38,8	866	40,2	1693	30,2	1482	37,9	616	40,5	446	34	8881	33,7
Migrate other country	277	5,9	512	10,9	342	13,8	269	12,5	599	10,7	331	8,5	138	9,1	107	8,2	2575	9,8
Total	4674	100	4692	100	2487	100	2154	100	5608	100	3911	100	1521	100	1310	100	26357	100
**Sent remittance somewhere**																		
No	3251	69,6	3541	75,5	1493	60	1276	59,2	4500	80,2	3382	86,5	992	65,2	940	71,8	19375	73,5
Yes	1423	30,4	1151	24,5	994	40	878	40,8	1108	19,8	529	13,5	529	34,8	370	28,2	6982	26,5
Total	4674	100	4692	100	2487	100	2154	100	5608	100	3911	100	1521	100	1310	100	26357	100

### Trends in quality-of-life scores

**Fig 2:**
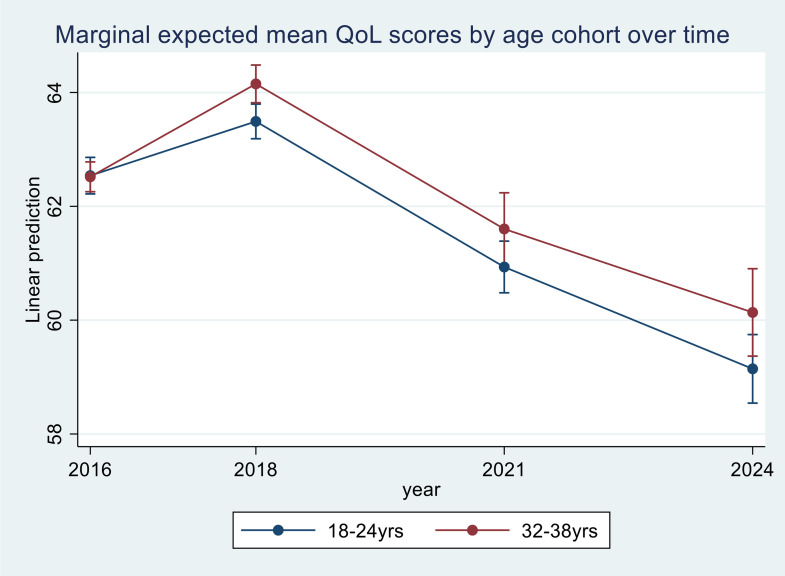
Expected marginal mean QoL scores for two age cohorts over 2016–2024 in Gauteng, South Africa.

In a mixed-effect model with district fitted as a random effect, adjusted for race, education, whether one was married or being in a relationship, household size, being in a household that received at least one social grant, respondent’s employment status, total household income, and type of housing, there was a significant interaction between time and age-group cohort variables, indicating a differential effect of QoL between the two age cohorts over time, Table 3. To show these differential effects, Table 4 and Fig 2 show the expected marginal mean scores for QoL for the two age cohort groups stratified by time (year). In 2016, the expected marginal mean QoL score for the age-cohort-1 (18–24-year-olds) was the same as that of the age-cohort-2 (32–38-year-olds), at 62.5. In the following survey in 2018, the expected marginal mean QoL score for age-cohort-1 increased to 63.5, while for cohort-2 the increase was higher, at 64.2. In 2021, the expected marginal mean QoL score for cohort-1 decreased by 2.6, from 2018 values, while for the same period, it decreased by the same absolute amount of 2.6 for cohort-2. From 2021 to 2024, the expected marginal means further decreased to 59.2 and 60.1 for cohort-1 and cohort-2 respectively.

**Table 3:** Quality of Life changes between 2016 and 2024 for two age cohorts staying in Gauteng.

	Model 1				Model 2			
	Uni-variable				Multi-variable			
	Regression Coefficient	p-value	95% CI		Regression Coefficient	p-value	95% CI	
**Time x Cohort interaction model**								
2016#18–24yrs	62,23	0,00	61,02	63,44	Reference			
2016#32–38yrs	−0,28	0,34	−0,86	0,29	0,08	0,64	−0,25	0,41
2018#18–24yrs	−0,44	0,30	−1,27	0,40	1,03	0,00	0,46	1,60
2018#32–38yrs	−2,78	0,00	−4,11	−1,45	−1,56	0,01	−2,70	−0,42
2021#18–24yrs	−3,45	0,00	−4,73	−2,16	−3,22	0,00	−3,91	−2,53
2021#32–38yrs	2,19	0,01	0,53	3,85	1,66	0,00	0,68	2,63
2024#18–24yrs	−1,26	0,00	−1,56	−0,97	−0,84	0,00	−1,26	−0,42
2024#32–38yrs	−1,82	0,00	−3,07	−0,58	−2,01	0,01	−3,62	−0,40
**Sex**								
Male	61,13	0,00	59,89	62,36				
Female	−0,47	0,01	−0,82	−0,13				
**Race**								
White	71,47	0,00	69,69	73,25	Reference			
Black African	−12,20	0,00	−13,44	−10,96	−2,88	0,00	−3,51	−2,26
Coloured	−6,83	0,00	−7,91	−5,75	−2,43	0,00	−2,79	−2,06
Indian/Asian	−3,87	0,00	−6,00	−1,74	−2,13	0,00	−3,29	−0,97
Other	−11,48	0,00	−12,52	−10,45	−3,97	0,00	−4,91	−3,04
**Education**								
Graduate or higher	73,12	0,00	71,82	74,43	Reference			
No education	−22,96	0,00	−24,15	−21,77	−11,77	0,00	−13,34	−10,20
Primary	−20,78	0,00	−21,59	−19,97	−8,81	0,00	−9,76	−7,85
Some secondary	−17,00	0,00	−17,74	−16,26	−6,90	0,00	−7,57	−6,23
Matric	−11,69	0,00	−12,35	−11,04	−5,11	0,00	−5,50	−4,72
Certificate/Diploma	−5,07	0,00	−5,59	−4,54	−2,27	0,00	−2,45	−2,09
Not Specified	−7,65	0,00	−10,03	−5,27	−4,05	0,00	−5,04	−3,07
**Relationship status**								
In a relationship	61,55	0,00	60,31	62,78	Reference			
Not in a relationship	−2,84	0,00	−3,55	−2,14	−1,24	0,00	−1,68	−0,81
**Household size**								
Four	63,11	0,00	61,21	65,02	Reference			
One	−4,90	0,00	−6,13	−3,67	−1,65	0,00	−2,49	−0,81
Two	−2,60	0,00	−3,52	−1,68	−1,12	0,00	−1,83	−0,41
Three	−1,50	0,00	−2,33	−0,67	−0,11	0,69	−0,64	0,42
Five	−0,77	0,14	−1,79	0,25	−0,04	0,90	−0,61	0,54
Six or more	−2,87	0,00	−4,25	−1,49	−0,36	0,42	−1,22	0,50
**At least one household memberr receives a social Grant**								
No	62,83	0,00	61,54	64,12	Reference			
Yes	−4,80	0,00	−5,60	−3,99	−0,85	0,00	−1,00	−0,69
Employment Status								
Full time employed	65,20	0,00	63,84	66,56	Reference			
Unemployed	−7,86	0,00	−8,51	−7,22	−2,63	0,00	−3,10	−2,16
Student	1,94	0,00	1,20	2,68	2,71	0,00	2,21	3,21
Retired/pension	−4,89	0,00	−6,79	−2,99	−1,14	0,06	−2,31	0,03
Business owner	−4,04	0,00	−4,84	−3,24	−1,25	0,00	−1,59	−0,90
Part-time employed	−7,73	0,00	−8,26	−7,20	−1,83	0,00	−2,22	−1,45
**Total monthly household income**								
R19201 or more	72,55	0,00	71,25	73,84	Reference			
No Income, < R1 600	−19,34	0,00	−20,33	−18,35	−7,94	0,00	−8,84	−7,05
R1601 - R6400	−15,05	0,00	−16,07	−14,04	−5,78	0,00	−6,41	−5,15
R6401 - R12800	−10,21	0,00	−11,63	−8,79	−4,35	0,00	−5,29	−3,41
R12801 - R19200	−5,24	0,00	−6,28	−4,19	−2,39	0,00	−2,98	−1,80
Prefer not to say	−7,99	0,00	−8,73	−7,26	−3,64	0,00	−4,26	−3,03
Don’t know	−11,17	0,00	−12,00	−10,34	−4,84	0,00	−5,66	−4,01
**House ownership**								
Owned	62,71	0,00	61,25	64,17				
Renting	−0,85	0,39	−2,77	1,07				
Allowed to stay	−5,72	0,00	−6,83	−4,61				
Squatting or liv	−19,77	0,00	−21,57	−17,98				
Other	−11,59	0,00	−13,50	−9,68				
Don’t know	−1,37	0,06	−2,78	0,05				
**Type of housing**								
Complex/Estate	68,11	0,00	66,73	69,49	Reference			
brick or concrete	−4,00	0,00	−5,12	−2,87	−0,77	0,01	−1,32	−0,22
Room/Flat or tent	−8,44	0,00	−9,30	−7,59	−3,61	0,00	−4,25	−2,96
Informal	−23,01	0,00	−24,07	−21,95	−15,76	0,00	−16,58	−14,94
**Migration status**								
Born in Gauteng	62,77	0,00	61,13	64,42				
Migrated internal	−4,07	0,00	−5,68	−2,46				
Migrated international	−4,81	0,00	−5,61	−4,00				
**Remittance**								
No	61,34	0,00	59,86	62,82				
Yes	−1,60	0,00	−2,69	−0,51				
**Intercept (Baseline)**					80,44	0,00	79,61	81,27

**Table 4:** Changes in QOL scores among two cohorts aged 18-24 years and 32-38-year-olds in 2016 over four rounds of QOL surveys 2016 - 2024 in Gauteng, South Africa.

		Delta-method				
	Margin	Standard error	t	P > t	95% confidence	interval
**year#cohort**						
**2016#18–24yrs**	62,54	0,16	381,83	0,00	62,22	62,86
**2016#32–38yrs**	62,52	0,13	468,60	0,00	62,26	62,78
**2018#18–24yrs**	63,49	0,16	408,83	0,00	63,19	63,80
**2018#32–38yrs**	64,15	0,17	379,50	0,00	63,82	64,48
**2021#18–24yrs**	60,93	0,23	263,28	0,00	60,48	61,39
**2021#32–38yrs**	61,60	0,32	190,06	0,00	60,97	62,24
**2024#18–24yrs**	59,15	0,31	192,59	0,00	58,54	59,75
**2024#32–38yrs**	60,14	0,39	153,44	0,00	59,37	60,90

**Fig 3:**
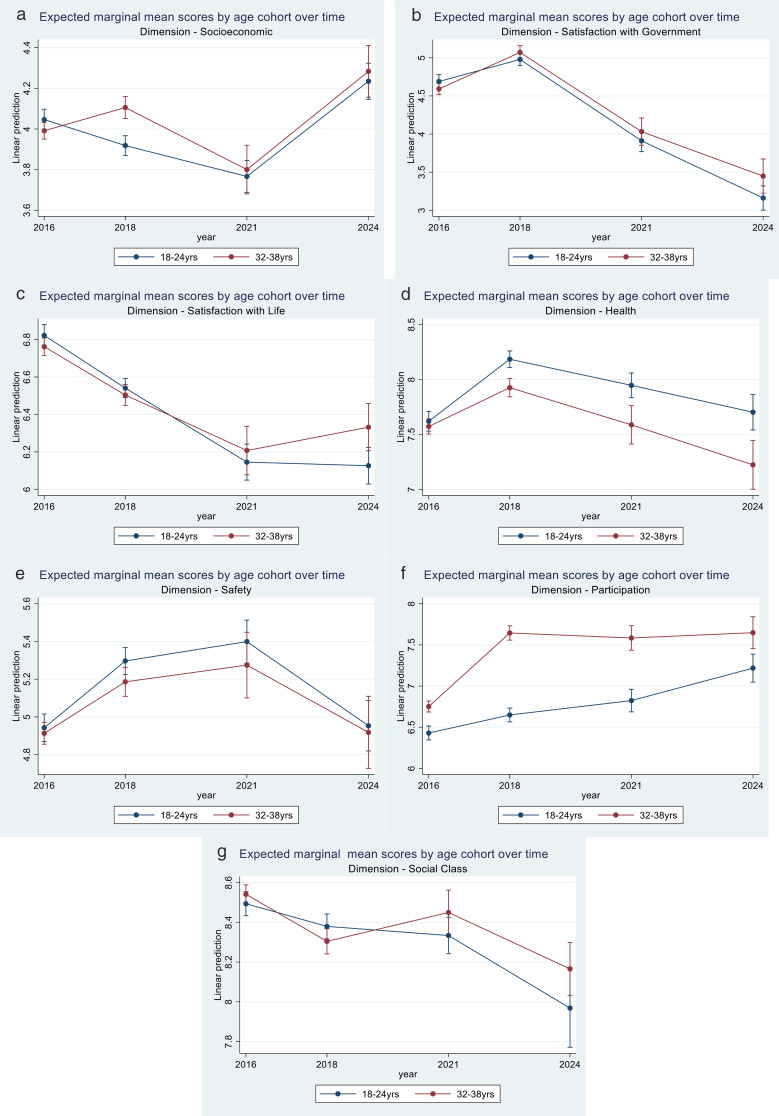
Expect marginal mean score for individual QoL dimensions for two age cohorts over time in Gauteng South Africa, 2016 - 2024.

To assess which dimension of the QoL scores had the most impact on changes in the overall QoL scores, adjusted for the same socio-demographic factors as used in the QoL model, multi-variable mixed effects regression models for each of the seven dimensions were fitted, and the expected marginal means for each age cohort group, time and interaction terms were estimated and plotted, Fig 3.

#### Socioeconomic status.

Marginal mean scores for the socioeconomic dimension for cohort-1 decreased between 2016 and 2021 before increasing in 2024, i.e., a U-shape trend, Fig 3a. In 2018, age-cohort-2 had a statistically higher expected marginal mean social class score than age-cohort-1, 4.1 vs. 3.9, but there was no evidence for a statistical difference between their marginal means in 2021 and 2024.

#### Satisfaction with government.

The marginal mean scores for the satisfaction with government performance dimension resembled those of the overall QoL scores, Fig 3b. In 2016, the marginal mean score for age-cohort-1 was 4.7, while that for age-cohort-2 it was 4.6, and in 2018, the mean scores for the two groups were 5.0 and 5.1, respectively. The marginal mean scores dropped to 3.9 and 4.0 for age-cohort-1 and 3.2 and 3.4 for age-cohort-2 in 2021 and 2024, respectively.

#### Life satisfaction.

Marginal mean scores for the life satisfaction dimension decreased for both groups between 2016 and 2021, and where similar, with over-lapping confidence intervals, Fig 3c. In 2024, marginal mean scores were 6.1 and 6.3 for cohort-1 and cohort-2 respectively.

#### Health status.

For the health dimension, marginal mean scores for the two age cohort groups showed similar trends, initially increasing between 2016 and 2018 but decreasing in 2021 and 2024 for both groups, although at a higher rate (steep gradient) for the older age cohort-2 group, Fig 3d. Mean health scores were consistently higher for the younger age-cohort-1 than the older group.

#### Safety.

Marginal mean scores for the safety dimension showed an inverted U-shape, increasing between 2016 and 2021 but subsequently coming down in 2024 to similar levels observed in 2016, Fig 3e. The age-cohort-1 group, with marginal mean safety scores of 4.9, 5.3, 5.4, and 5.0 for 2016, 2018, 2021, and 2024, respectively, had consistently higher scores than the age-cohort-2 group, with scores of 4.9, 5.2, 5,3 and 4.9 for the four time periods.

#### Participation.

Marginal mean scores for the participation dimension for age-cohort-1 increased monotonically over the four survey rounds from 6.4, 6.6, 6.8, and 7.2 for 2016, 2018, 2021, and 2024, respectively, Fig 3f. For the age cohort-2 group, the marginal mean score for participation increased from 6.8 to 7.6 between 2016 and 2018, remaining at 7.6 units in 2021 and 2024.

#### Services.

The marginal mean scores for services were similar over the study period, with overlapping confidence intervals across comparison groups, Fig 3g.

### Other factors associated with quality-of-life scores

Among other factors associated with QoL in an adjusted multi-variable model (Table 3, Model 2) was race, where the marginal mean scores for Black African 2.88 units lower, Coloureds, 2.43 units lower, Indian/Asian, 3.13 units lower, were all statistically lower than that of Whites. There was no evidence of a statistical difference between Coloureds, Indian/Asian, and Black African race groups in QoL mean scores. In the same model, QoL scores monotonically increased with the level of education, with those with graduate or post-graduate education having the highest QoL, and those with no education with the lowest, i.e., 11.77 units below those with graduate or post-graduate qualifications. Those who were single, divorced or widowed had a 1.24 lower QoL marginal mean score than those reporting being married or in a relationship. Participants living in households made up of three people or more reported a better quality of life than those living alone. Participants living in households that received at least one social grant had a 0.85 lower expected mean QoL score compared to those who did not. Compared to those who were in full time employment, those who were unemployed, – 2.63, those in part-time employment, – 1.83, those who reported running a business, – 1.25 and those who were in retirement, – 1,14 all reported lower mean scores. Like education, QoL increased monotonically increased with total household income. Compared to those who stayed in complexes or estates (“affluent gated communities”), those living in informal housing had a 15.76 units lower mean, while those who said they rented a room or flat or stayed in a tent had a 3.61 lower score.

## Discussion

In this study, young people aged 18–24 in Gauteng province reported progressive declines in their quality of life since 2016. This decline was not unique to that group, as similar decreases were also observed among the 32- to 38-year-old age comparison group. However, the QoL scores of the older age group remained consistently higher compared to the younger age group. This drop in QoL indicates systemic failure in solving the root causes of discontent among young people, further aggravated by the residual socio-economic consequences of the COVID-19 pandemic. This decay presages an up-and-coming vulnerability in socio-economic status, health, and safety domains that need urgent policy intervention.

Historical factors such as economic exclusion due to apartheid, education inequality, and systemic underinvestment in youth development continue to shape current realities in South Africa, and this continue to affect the youth. Young people in South Africa face a multitude of challenges that include high levels of unemployment, economic inequalities, high rates of crime, alcohol and substance abuse, violence, anxiety, depression, and trauma [12,40–42]. The decline and observed changes across different dimensions of QoL among the study group reflects the inter-connectedness and complex ways different aspects, factors and domains influence how people subjectively and/or objectively perceive their lives in line with the Schalock’s multidimensional Approach [32].

The findings are consistent with global trends, where youth in transitional economies experience a heightened risk of unemployment, social exclusion, and reduced access to essential services [43]. In South Africa, youth unemployment remains disturbingly high, with estimates above 60% for people aged 15–24 years in recent years [44]. The resulting economic stagnation limits their potential for upward mobility and increases conditions of mental health problems, anxiety, depression, and trauma common in this population.

Contributing to the decline in overall QoL among young people in our study was a consistent decline in satisfaction with the life dimension over the eight-year study period. Interestingly, for the older age cohort, although there was a noticeable decline in satisfaction on the life dimension between 2016 and 2021, there were signs that this had started to improve, probably indicating general economic and social improvements after the COVID-19 pandemic [45,46]. The increasing gap in QoL scores between the two groups in the study underlines more clearly the growing disparities in the population. It also reflects that the COVID-19 recovery efforts were not equitably observed, as this marginal improvement in socio-economic status did not benefit the younger cohort. This again reinforces the urgent need for targeted social and economic policy interventions, targeting vulnerable young people, particularly those from marginalised communities.

Worryingly, though, despite an initial increase in the health dimension scores among young people between 2016 and 2018, there was a noticeable decline in health scores from 2018 to 2024. This trend was also observed among the older age cohort, with scores for this group being consistently lower than the younger group. It is expected that young people would generally report better health, so this consistent decline in health scores is a disturbing phenomenon. It is not clear how much of this decline is associated with mental health, but this has been reported as one of the challenges faced by young people [47]. Although there is a lack of data on the burden of depression and mental health among young people, anecdotal evidence suggests that there may be high levels of depressive and mental health conditions in this population group [48]. According to the UNICEF South Africa U-Report poll results (2023), more than 60% of young people needed mental health support [49]. COVID-19 probably worsened the mental health of young people, as demonstrated by a study in China that showed that 14.4% of sampled youth experienced post-traumatic stress disorder (PTSD) [50]. In our study, the health dimension scores declined much higher for the older age group than the younger cohort. By the 2024 survey, some of the older cohorts were in their mid-forties, some of the decline in health may be explained by the increasing incidence of chronic conditions associated with ageing [51,52].

The services dimension saw a consistent decline among young people, while there was a slight uptick among the older age group, possibly reflecting the difference in socio-economic conditions of the two groups. According to the Governance, Public Safety and Justice Survey (GPSJS 2022/23), the percentage of individuals who rated public services as satisfactory had declined in nine out of twelve government services assessed between 2019/20 and 2022/23 [53]. The nine service areas that saw a decrease included the education theme that covered public schools and higher learning; the justice, crime prevention, and security theme that covered courts and the South African Police Services (SAPS); and the “other services” theme that covered the South Africa Social Security Authority (SASSA), South Africa Revenue Services (SARS) and Home Affairs department services. In that survey, the services that saw an improvement in satisfactory rating included the public health theme, which covered public hospitals and clinics, and the justice, crime prevention, and security theme, which covered correctional services.

The decline in satisfaction with the government’s performance mirrored the overall QoL score, thus indicating more disillusionment with how the government managed and implemented its policies, services, and programmes. This observation was similar to results observed in a study undertaken by Statistics South Africa (Stats SA), the Governance, Public Safety and Justice Survey (GPSJS 2022/23), which saw levels of trust in government institutions among participants declining in two-thirds (10 out of 15) of government institutions between the 2019/20 and 2022/23 surveys [53].

A look at the socio-economic dimension shows that, whilst socioeconomic status consistently declined between 2018 and 2021, it seemed to have picked up in 2024, reaching levels similar to those seen in 2016, possibly suggesting improvements and a positive outlook after the COVID-19 pandemic [54]. Several government-led programmes such as the Expanded Public Works Programme (EPWP), Community Work Programme (CWP), National Youth Service Programme, Entrepreneurship training programmes, National Youth Development Agency (NYDA), and the Presidential Young Employment Initiative are meant to develop skills and to provide work experience for youth, aimed at creating permanent employment opportunities. However, these have not necessarily reduced youth unemployment levels in the country, with a mismatch between skills needed in industry and the youthful workforce [55,56].

Among both cohorts, the safety domain saw an improvement between 2016 and 2021, but these had since dropped to levels seen in 2016. Whilst some initial increases could be partially explained by the lockdowns and restrictions during the height of the COVID-19 pandemic, returning to some form of “normal” meant fewer restrictions and less safe environments. According to a STATS SA report, Gauteng province reported a decline of 2.6% in overall reported crime, where rape decreased by 7.4% and sexual assault decreased by 20,5% in the third quarter of 2023 [57]. However, the province remains the epicentre of crime in the country, and according to the same cited report, murder increased by 2,9%, attempted murder increased by 8,9%, and common assault increased marginally by 0.8%. Being exposed to crime indirectly affects young people’s mental health due to stress and anxiety [58]. Various surveys, including the Social Attitudes Surveys and past QoL Surveys, have placed crime as one of the top issues people worry about in the province and the country [59,60]. The STATS SA study mentioned above reported increases in six out of seven crime-reported domains: housebreaking with a 6% increase, household robberies increased by 18%, assault with a 3% increase, theft of motor vehicles increased by 28%, murder by 43% and sexual offences increased by 53%. In the same study, deliberate damage to property was the only category that decreased, which saw a decline of 16% between 2019/20 and 2022/23 [53]. Gauteng province had the highest percentage of individuals experiencing theft of personal property, and a high proportion of respondents also indicated that they did not feel safe walking alone at night.

In our study, the only dimension that consistently increased over the entire eight-year study period among young people was the ‘participation’ domain. This can be interpreted in several ways, including that young people are increasingly becoming interested in how things are run in their province, thus the increase in participation, or that they are so disillusioned that they find it necessary to be more engaged and involved in matters that affect them. However, this increase in the participation dimension gives cause for hope, as young people, despite their challenges, remain engaged in civic and community activities. This resilience is another sign that the desire to be given a space to contribute meaningfully to decision-making processes among youth is essential. Policies that draw on this participatory potential may increase agency and belonging, blunting some of these other negative trends.

When young people finish school, some acquire skills and enter the job market. There is a reported skills shortage in specific sectors of the economy. Yet, there is high unemployment among this age group, even among young people who hold formal post-matric qualifications. There are indications that skills training is not in line with what is needed in the economic market [61,62]. The proportion reporting some formal training among the young age group in our study plateaued at 34% in 2024, thus indicating that the majority of this age group may be unskilled and might find it difficult to find formal employment. Some service delivery challenges in the province are related to insufficient artisanal skills and/or the right quantity of skilled workers required to improve public services. There is a need for Technical and Vocational Education and Training (TVET) colleges to offer appropriate training, and for trained young people to be absorbed into local governments and municipalities [63,64].

The fact that young people send money (remittances) to families and/or relatives indicates the enormous economic challenges faced by this group and their families. It is unlikely that recent school and/or college graduates earn high salaries; the median monthly national income in 2022 was ZAR 5 200 (US$297), and ZAR 4 300 (US$246) among young people [65]. These are relatively low salaries, showing possible financial and emotional strain on the population, especially among this young age group. According to the last Living Conditions Survey (2015/6), nearly half of South African adults lived below the lower poverty line [66]. Although Gauteng was cited as one of two provinces in the country with the lowest number of adults living below the poverty line, 29% of adults were still living below the poverty line. Many of the young cohort group respondents came from a household where at least one person received a government social grant, showing high levels of poverty [66,67].

The decline in overall QoL, with contrasting changes in individual QoL dimensions, that included consistent declines in life, services and government performance dimensions, inverted U shapes in health, and safety, a U shape in the socio-economic dimension and a consistent increase in the participation dimension reflects the complex, non-linear nature of how individuals perceive their lives [32]. The dimensions that were explored in this study are in line with Schalock’s Multidimensional Approach’s interconnectedness of factors that affect people’s lived experiences. Study participants’ responses to the survey questions are likely influenced by a combination and overlap of different factors. For example, education levels likely influence if a person gets a job, what kind of job they get and how much they will earn (an economic dimension), while unemployment, economic exclusions are likely to be associated with mental health (psychological dimension), which in turn will likely impact relationships with family and friends (a social dimension). On the other hand, alcohol abuse, substance use (psychological domain), and provision of services by government such as street lighting (a governance and political dimension) are likely to be associated with community safety issues (an environmental dimension). In addition to these spatial factors, there is also the temporal dimension which impacts on how individuals view and rate their QoL, for example how people perceived their quality of life before, during and after the COVID-19 epidemic was likely to be influence by the stage of the event.

### Limitation

Quality-of-Life measures use both subjective and objective measures. Subjective assessments are influence by past and current experiences as well as perceptions about the future, thus significant personal experiences and/or national events such as the COVID-19 outbreak in 2019/2020 are likely to have had an impact on reported quality-of-life measure in this study. Although the study assumes a ‘cohort’ approach, study participants in each round of the survey were randomly selected at each round, making this study a series of independent cross-sectional surveys over time. The surveys did not collect information on participation in previous surveys from participants; thus, the study could not ascertain the number of participants who participated in multiple surveys.

## Conclusion

Young people face multiple challenges, including limited economic opportunities and disillusionment with life. Their discontentment in South Africa is growing, and this needs to be urgently addressed. The decline in satisfaction with public services and governance further highlights a breakdown in trust between young people and state institutions. This dissatisfaction has far-reaching implications for social cohesion and stability, as seen in the recent widespread unrest in South Africa and Kenya, where socio-economic grievances and perceptions of government inefficiency were key drivers of unrest [68–71]. Without concerted efforts to restore trust and deal with these systemic inequities, there is a strong possibility of recurring unrest [68].

Growing discontent among South African youth is a multidimensional policy challenge. Interventions should be directed toward unemployment, education, health, and other areas that improve socioeconomic conditions. Beyond this, efforts must be made toward inclusive economic recovery strategies that put the interests of young people at the forefront if the downward trend in their living standards is ever to be reversed. Failure to act decisively risks perpetuating cycles of unrest and instability, with severe implications for the country’s socio-economic future. Most young people have great aspirations, with high hopes of assuming leadership positions, aspiring to be economically active, and meaningfully contributing to the country’s development.
